# Expression of spike and hemagglutinin-esterase proteins is necessary to recover infectious recombinant bovine coronavirus

**DOI:** 10.1128/jvi.01027-25

**Published:** 2025-08-11

**Authors:** Yoshiro Sugiura, Tatsuki Takahashi, Shiori Ueno, Sodbayasgalan Amarbayasgalan, Kenta Shimizu, Makoto Ujike, Tohru Suzuki, Wataru Kamitani

**Affiliations:** 1 Department of Infectious Disease and Host Defense, Graduate School of Medicine, Gunma University 12925, Maebashi, Gunma, Japan; 2 Laboratory of Veterinary Infectious Diseases, Faculty of Veterinary Medicine, Nippon Veterinary and Life Science University 12989, Musashino, Tokyo, Japan; 3 Research Center for Animal Life Science, Nippon Veterinary and Life Science University 12989, Musashino, Tokyo, Japan; 4 Division of Zoonosis Research, Sapporo Research Station, National Institute of Animal Health, NARO 73954, Sapporo, Hokkaido, Japan; Emory University School of Medicine 12239, Atlanta, Georgia, USA

**Keywords:** BCoV, reverse genetics, reporter virus

## Abstract

Bovine coronavirus (BCoV) is a significant pathogen in cattle, and its virological analysis has been hampered by the difficulty in isolating and culturing field isolates with high titers. Here, we successfully generated recombinant BCoV using an infectious bacterial artificial chromosome DNA clone co-expressing the BCoV spike (S) and hemagglutinin-esterase (HE) proteins. We also investigated the role of trypsin in BCoV culture using a recombinant virus expressing the ZsGreen reporter gene (Rec-BCoV-Kakegawa-ZsGreen). We found that an optimized concentration of 2.5 µg/mL significantly enhanced viral titers, reaching 2 × 10^4^ TCID_50_/mL. To explore the functional significance of the ORF2 protein, we engineered a recombinant virus in which the ORF2 gene was replaced with ZsGreen. While Rec-BCoV-Kakegawa-ZsGreen exhibited growth kinetics comparable to those of the parental BCoV Kakegawa strain during early infection, peak titers were lower, suggesting a possible role for the ORF2 protein in the later stages of the viral replication cycle. Additionally, we determined the role of S and HE protein expression in the recovery of recombinant BCoV from infectious DNA using the ZsGreen virus. In HE or S protein alone, the signal of the reporter protein ZsGreen in transfected cells was stronger than that of infectious DNA alone, but no infectious virus particles were recovered in subsequent steps. However, infectious viral particles were successfully produced only when both HE and S were present. This indicates that the addition of S and HE is necessary to produce recombinant BCoV, and the present method provides important insights into the replication mechanism and pathogenicity of BCoV.

**IMPORTANCE:**

In this study, we generated a recombinant BCoV (Rec-BCoV-Kakegawa-WT) using an infectious bacterial artificial chromosome DNA clone and confirmed that the HE protein enhanced viral release. We also identified that an optimal trypsin concentration (2.5 µg/mL) improves viral titers. Additionally, we developed a reporter virus with a ZsGreen insertion, suggesting that the ORF2 protein may play a role in late-stage viral replication. This study contributes to the optimization of BCoV culture conditions and advances vaccine development.

## INTRODUCTION

Bovine coronavirus (BCoV) has been identified as the causative agent of respiratory and enteric disease in cattle. BCoV belongs to the family *Coronaviridae*, genus *Betacoronavirus,* and subgenus Embercovirus (1). BCoV causes economic losses in the cattle industry owing to mortality in calves and reduced growth performance and milk production in feedlots and dairy cattle. BCoV is antigenically and genetically similar to the human coronavirus OC43 (HCoV-OC43), which belongs to the species Betacoronavirus1 and is considered a host-range variant (2, 3).

BCoV possesses an approximately 31 kb viral genome in virus particles. ORF1ab, which occupies two-thirds of the viral genome, encodes 16 nonstructural proteins via a frameshift mechanism (4). These 16 nonstructural proteins include viral polymerase (RdRp) and virus-derived proteins involved in viral RNA synthesis. Similar to other coronaviruses, the remaining one-third of the viral genome encodes the spike (S), envelope (E), membrane (M), and nucleocapsid (N). The BCoV genome also encodes hemagglutinin-esterase (HE) structural proteins and contains genes of ORF2 (32 kDa protein) and ORF7 (12 kDa protein) whose functions are not well understood (1, 5).

Similar to other viruses, a reverse genetics system has been established for coronaviruses (6). First, recombinant viruses can be recovered by introducing RNA synthesized *in vitro* into cells by electroporation (7–11) or by inserting the full-length coronavirus into a bacterial artificial chromosome (BAC), which is excellent at retaining long DNA strands downstream of the cytomegalovirus promoter and then inserting the infectious DNA into cultured cells (12–17). Second, a method for using the vaccinia virus as a vector for coronavirus cDNA has been established (18, 19). The use of yeast-based systems is another option (20). In addition, PCR-based methods have been developed to avoid mutational insertions during cloning (21).

For HCoV-OC43, which is genetically related to BCoV, the generation of recombinant viruses using BAC has been reported (16, 22, 23). Recently, the generation of a reporter virus for HCoV-OC43 using yeast centromeric plasmids was reported. Specifically, this method uses an N-expression plasmid that is co-expressed with infectious YAC DNA when introduced into the cells (24). No report has yet been published on a reverse genetics system for BCoV, despite the fact that BCoV is genetically closely related to OC43.

Infectious DNA was constructed using the Gibson method, a seamless cloning method, with the Kakegawa strain, a laboratory strain, and BAC to generate infectious DNA. To simplify the detection of infected cells, infectious BAC DNA was constructed by replacing the ORF2 gene of bovine coronavirus with ZsGreen. DNA was then transfected into HRT-18G cells to recover the recombinant virus. This study identified a stable and efficient method for recombinant virus recovery, which involved the co-expression of S- and HE-expressing plasmids during transfection. Furthermore, this study analyzed the requirement of trypsin during the culture of bovine coronaviruses by utilizing a reporter virus to determine the required concentration of trypsin. The findings of this study will contribute to the development of an efficient method for generating recombinant bovine coronaviruses. In addition, trypsin requirements were determined using a reporter virus with a recombinant bovine coronavirus. The method established in this study can be applied to coronaviruses, including bovine coronaviruses, for which it is difficult to generate recombinant viruses.

## RESULTS

### Rescue of recombinant bovine coronavirus using an artificial bacterial system

In general, reverse genetics is a powerful tool for virology research. Similar to many other viruses, coronaviruses can be rescued from infectious DNA and RNA. To the best of our knowledge, there have been no reports on the creation of recombinant viruses from infectious DNA or RNA of bovine coronaviruses. We used a BAC-based reverse genetics system with co-expression of viral structural proteins of bovine coronaviruses.

**Fig 1:**
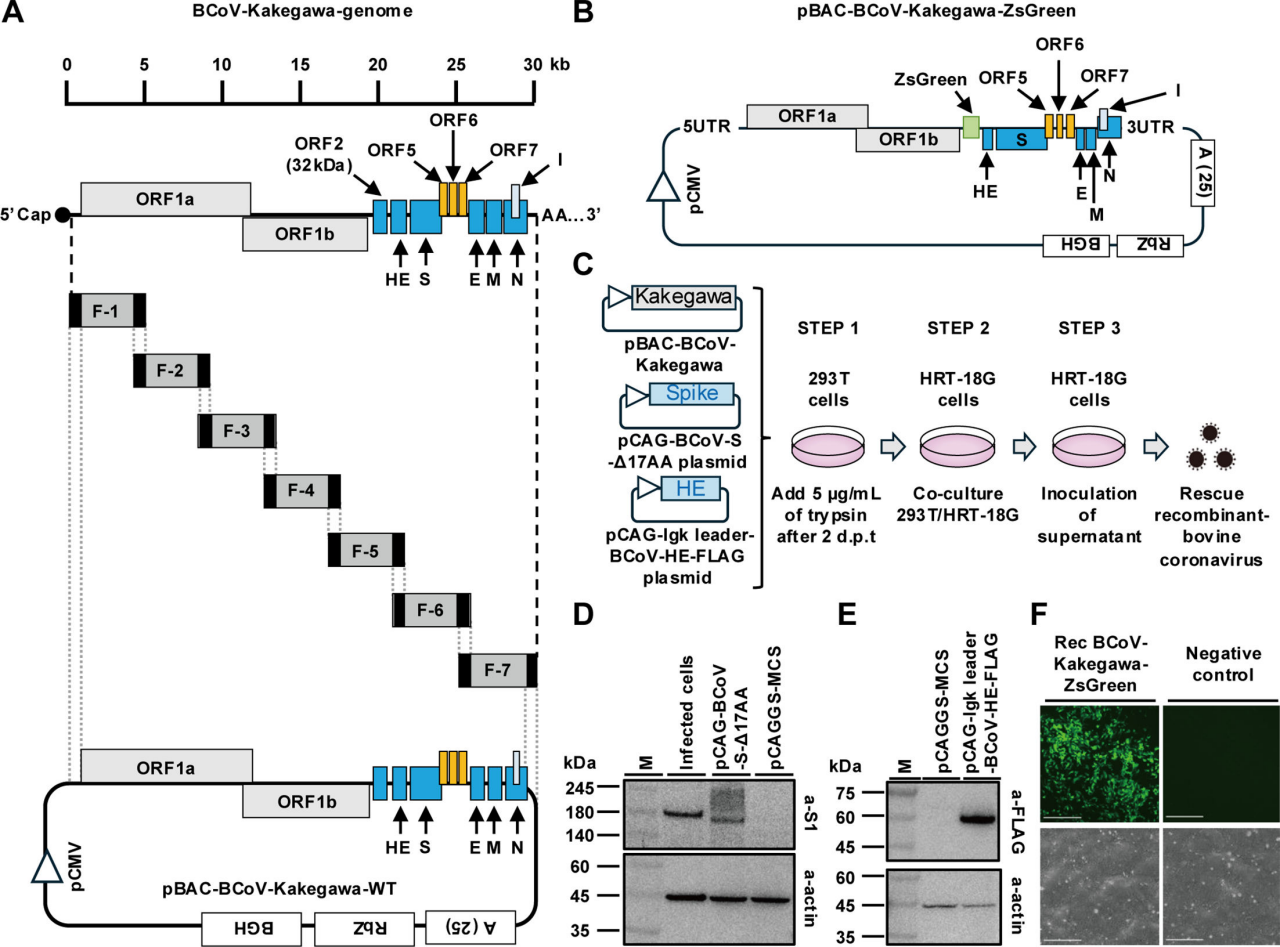
Establishment of a new reverse genetics’ method for recombinant bovine coronavirus. (**A**) Schematic diagram of the genome structure and cloning stratagem using Gibson assembly. The genome sequence of BCoV-Kakegawa-WT was divided into seven fragments. The individual fragments were amplified by using specific primer sets, as shown in Table S1. Each fragment has 40 overlapping nucleotide sequences at both the 5ʹ and 3ʹ ends (black boxes). The bottom is a schematic diagram of pBAC-BCoV-Kakegawa-WT. pCMV: cytomegalovirus promoter, 5UTR: 5ʹ untranslated region, ORF1a, ORF1b, ORF2 (32 kDa protein), ORF5, ORF6, ORF7: open reading frame 1 a, 1b, 5, 6, 7, HE: hemagglutinin esterase, S: spike, M: matrix, E: envelope, N: nucleoprotein, I: Internal protein, 3UTR: 3ʹ untranslated region, A(25): synthetic 25 nt polyA, RbZ: ribozyme sequence of HDV, BGH: bovine growth factor polyadenylation signal. (**B**) Schematic diagram of the construction of infectious cDNA clones carrying the full-length genome of the Kakegawa bovine coronavirus strain. Using the Red/ET recombination method, the ORF2 gene of the Kakegawa strain of bovine coronavirus was replaced with the ZsGreen gene. (**C**) Schematic diagram illustrating the procedure for producing recombinant bovine coronavirus using infectious BAC DNA. BAC DNA containing the ZsGreen gene was co-transfected with two expression plasmids, pCAG-BCoV-S-Δ17AA and pCAG-Igk leader-BCoV-HE-FLAG, into 293T cells (Step 1). On day 2 post-transfection, culture media were replaced with FBS-free DMEM containing 5 µg/mL of trypsin and overlaid with HRT-18G cells once the positive area of ZsGreen expanded (Step 2). After 2 days of co-culturing in the presence of 5 µg/mL trypsin, the cells were maintained in DMEM with 10% FBS in the absence of trypsin until the area of ZsGreen-positive cells expanded. Freeze-thawing was used to obtain the recombinant P0 virus (Step 3). The obtained P0 virus was inoculated into new HRT-18G cells, and the P1 virus was collected via freeze-thawing from the supernatant of the infected HRT-18G cells. Detection of S protein (**D**) and HE protein (**E**) in transfected 293T cells by Western blotting. Lysates of 293T cells transfected with the indicated plasmids were prepared 48 hours post-transfection and subjected to Western blotting using an anti-OC43-S1 polyclonal antibody (a-S1 Ab) (**D**), anti-DYKDDDK monoclonal antibody (a-FLAG Ab), or anti-actin antibody (a-actin) as the primary antibody. M, molecular weight marker; infected cells, lysate of HRT-18G cells infected with BCoV-Kakegawa strain, pCAGGS-MCS: empty vector. (**F**) Spread of ZsGreen fluorescence in recombinant virus from the supernatant of co-cultured HRT-18G cells. The supernatant of the co-cultured HRT-18G cells was used to inoculate new HRT-18G cells. After 5 days, the ZsGreen protein was observed under a fluorescence microscope (Thermo Fisher Scientific, M7000). The right panel represents the results of the same procedure as on the left, using untransfected 293T cells as a negative control. Scale bar = 275 µm.

To generate an infectious BAC-DNA with full-length BCoV (Kakegawa strain) and an untranslated region, cDNAs were synthesized using RNA extracted from HRT-18G cells infected with the Kakegawa strain of BCoV. To construct infectious BAC DNA using the Gibson Assembly Ultra kit, seven fragments with 40 nt homology sequence were amplified with the cDNAs as templates and using GXL polymerase (Takara). A BAC with a cytomegalovirus promoter, 25 nt synthetic polyA, HDV ribozyme, and BGH polyadenylation signal sequences was used as a backbone vector according to previous reports (13, 25). BAC with a 40 nt homology sequence was amplified by PCR and subjected to a Gibson assembly reaction with the seven amplified PCR products (Fig. 1A and Table S1). In addition, pBAC-BCoV-Kakegawa-ZsGreen, in which the ORF2 gene of the BCoV gene was replaced by the ZsGreen gene, was constructed using the Gibson assembly method for reporter virus generation, as shown in Fig. 1B. As shown in Table 1, the sequence of the constructed BAC DNA was confirmed by Sanger sequencing. Nineteen nonsynonymous genes were identified in the generated BAC DNA. No base insertions or deletions were observed, as shown in Table 1.

**TABLE 1:** Mutations in pBAC BCoV Kakegawa WT

Mutation no.	Nucleotide position	Gene	Nucleotide mutation	Amino acid mutation
1	408	1a (nsp1)	T → C	Synonymous
2	1031	1a (nsp1)	G → A	R → K
3	1392	1a (nsp1)	T → A	H → Q
4	2424	1a (nsp2)	T → C	Synonymous
5	2700	1a (nsp2)	T → C	Synonymous
6	3248	1a (nsp3)	C → A	A → D
7	3752	1a (nsp3)	G → A	G → E
8	7746	1a (nsp3)	T → C	Synonymous
9	9160	1a (nsp4)	C → T	L → F
10	12887	1a (nsp9)	C → T	S → F
11	14186	1b (nsp12)	C → T	Synonymous
12	15756	1b (nsp12)	A → G	M → V
13	15807	1b (nsp12)	C → T	A → V
14	18924	1b (nsp14)	T → C	C → R
15	21486	1b (nsp16)	G → A	V → I
16	22625	HE	G → A	G → S
17	22748	HE	G → A	V → L
18	23759	S	C → T	T → I
19	24398	S	A → C	Y → S
20	25231	S	G → A	D → N
21	26593	S	T → A	S → T
22	27157	S	C → A	P → T
23	28522	E	C → G	L → V
24	28641	E	C → T	Synonymous
25	28729	M	C → G	T → S
26	28806	M	A → G	I → V
27	28820	M	T → C	Synonymous
28	28898	M	C → T	Synonymous
29	28961	M	C → T	Synonymous
30	29231	M	C → T	Synonymous
31	29241	M	T → C	F → L
32	29279	M	T → C	Synonymous
33	29380	M	T → A	I → N
34	29381	M	A → T	I → N
35	29542	N	C → T	A → V
36	29618	N	A → G	Synonymous
37	29624	N	G → A	Synonymous
38	29627	N	T → G	D → E
39	29675	N	G → A	Synonymous
40	29720	N	T → C	Synonymous
41	29751	N	C → T	Synonymous
42	29819	N	T → C	Synonymous
43	29882	N	C → T	Synonymous
44	30558	N	A → G	N → D

Next, we transfected pBAC-BCoV-Kakegawa-ZsGreen into 293T cells. Transfected cells were incubated until a ZsGreen signal was observed under a microscope. However, the ZsGreen signal in infected cells did not increase. Consequently, we co-transfected pBAC-BCoV-Kakegawa-ZsGreen together with the S and HE proteins of the BCoV expression plasmids into 293T cells. The signal of ZsGreen in the 293T cells treated with 5 µg/mL of trypsin increased (Fig. 1C, step 1). Subsequently, HRT-18G cells, a standard cell line for BCoV cultivation, were overlaid onto trypsin-treated 293T cells. The co-cultured cells were then incubated in the presence of 5 µg/mL of trypsin until the ZsGreen signal was sufficiently observable under microscopic analysis (Fig. 1C, step 2); the cells in the media were frozen and thawed and then centrifuged to remove debris. The collected supernatant was stored as a P0 virus. To confirm the infectious capability of the P0 virus and recombinant BCoV-expressing ZsGreen, the P0 virus was inoculated into naïve HRT-18G cells (Fig. 1C, step 3). As shown in Fig. 1F, ZsGreen signals were observed in naïve HRT-18G cells infected with P0 recombinant BCoV-Kakegawa-ZsGreen virus.

Following the construction of expression plasmids for S and HE proteins, their expression levels were confirmed by Western blotting. The expression of both proteins was observed in infected 293T cells, as shown in Fig. 1D and E. Although the VSV pseudotype virus system and the reverse genetic system of BCoV are different, we constructed an S protein expression plasmid from the BCoV Kakegawa strain with a 17 amino acid deletion at the C-terminus, as reported by Hulswit et al. (26). Expression of the S protein with a deletion of 17 amino acids was observed at a position slightly lower in molecular weight than that of the S protein derived from infected cells in Fig. 1C. Given the role of the HE protein in BCoV in depleting intracellular and cell-surface 9-O-Ac-Sia pools—an essential step for enhancing the production of pseudo-type VSV with BCoV-S (26)—we also assessed the co-expression of the HE protein. Western blotting confirmed the expression of the HE protein in infected 293T cells in Fig. 1E. The results of our study demonstrated that the recombinant reporter BCoV expressing ZsGreen was successfully rescued from co-cultured cells expressing both S and HE proteins.

### Trypsin requirement in BCoV replication in culture cells

**Fig 2:**
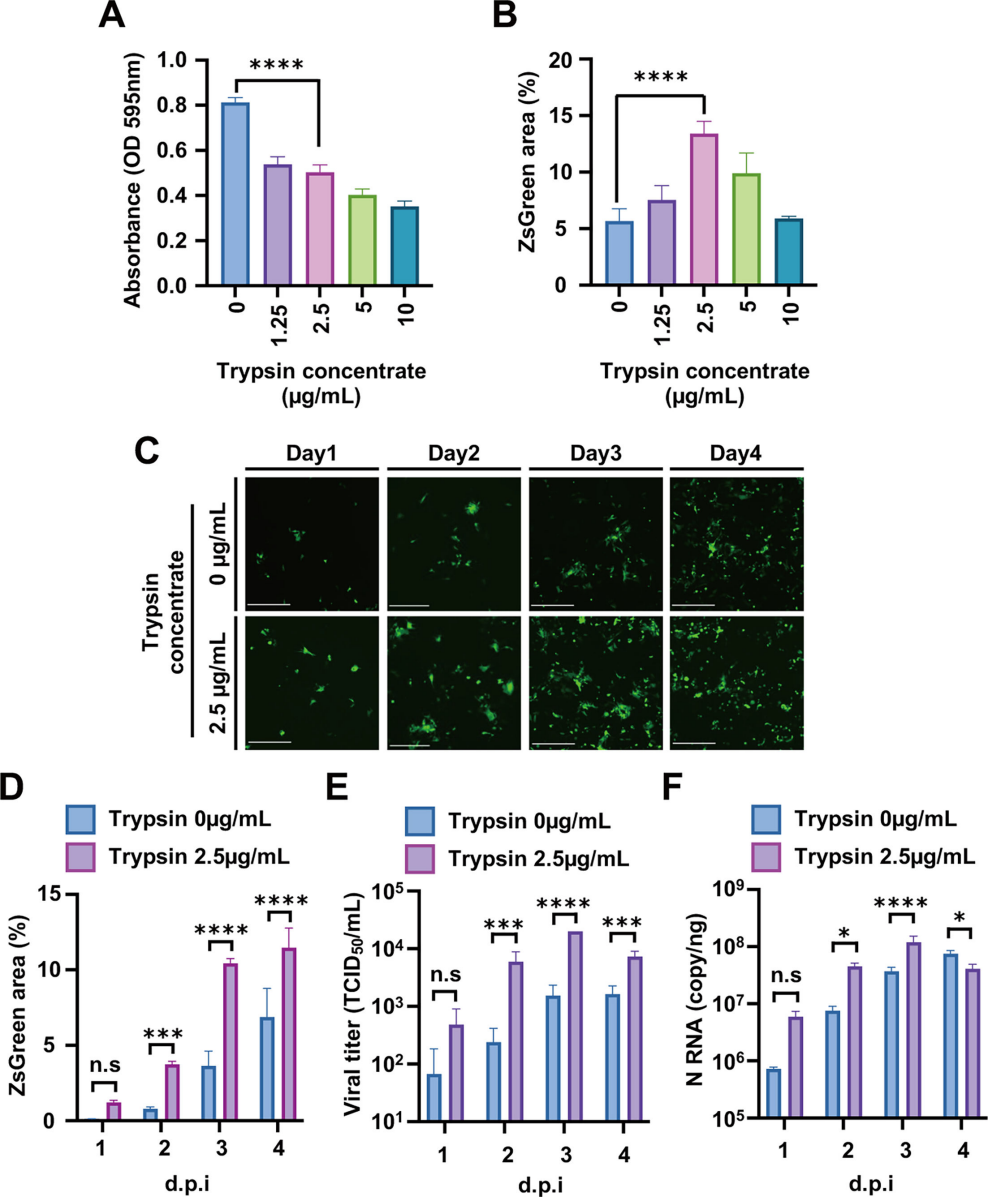
Trypsin requirement for viral replication of Rec-BCoV-Kakegawa-ZsGreen. Determination of a suitable trypsin concentration for HRT-18G cells (**A**) and replication of BCoV (**B**). Cell viability was determined using the MTT assay kit (Nacalai Tesque) at the indicated time points without BCoV infection (**A**). HRT-18G cells were infected with Rec-BCoV-Kakegawa-ZsGreen at an MOI of 0.001, and the infected cells were cultured in the presence of the indicated concentration of trypsin. Then, the spread area of ZsGreen protein was calculated using ImageJ software (**B**). The Y-axis represents the absorbance at 594 nm from the results of the MTT assay (**A**) and ZsGreen-positive regions as a percentage (**B**). The values represent the means ± SD from three independent experiments. ****P < 0.0001. (**C**) Time-dependent increase in ZsGreen protein levels in HRT-18G cells infected with Rec-BCoV-Kakegawa-ZsGreen in the presence of trypsin. Images were acquired using an M7000 microscope. Scale bar = 275 µm. (**D**) Quantification of ZsGreen fluorescence area for Rec-BCoV-Kakegawa-ZsGreen for 1–4 d.p.i. ZsGreen fluorescence was captured as the tile image and quantified using ImageJ. The values represent the means ± SD from three independent experiments. ***P < 0.001. ****P < 0.0001. Growth kinetics of infectious Rec-BCoV-Kakegawa-ZsGreen in culture medium (**E**) and accumulation of viral RNA in infected cells (**F**). Rec-BCoV-Kakegawa-ZsGreen was inoculated into HRT-18G cells at a multiplicity of infection (M.O.I) of 0.001, and the medium from these infected cells was collected at the indicated time points. The infectivity of the collected medium was determined using the tissue culture infectious dose (TCID50) method (**E**). Rec-BCoV-Kakegawa-ZsGreen was inoculated into HRT-18G cells, and the amount of N RNA was determined by one-step real-time PCR (**F**). ns, no statistically significant difference; d. p. i., days post-infection. The values represent the means ± SD from three independent experiments. *P < 0.05, ***P < 0.001, and ****P < 0.0001.

In the following analysis, the need for trypsin in the cultured cells for BCoV was investigated using the recombinant BCoV-Kakegawa-ZsGreen virus. HRT-18G cells were washed twice with 1× PBS. Subsequently, the recombinant BCoV-Kakegawa-ZsGreen was inoculated into pre-washed HRT-18G cells. Following a 1 hour incubation at 37°C, the cells were washed with 1× PBS once and then incubated with fetal bovine serum (FBS)-free Dulbecco’s modified minimum essential medium (DMEM) containing the indicated trypsin concentration (Fig. 2B). Although a cytotoxic effect was observed in the presence of 10 µg/mL trypsin (Fig. 2A), the ZsGreen signal increased to a 2.5 µg/mL concentration of trypsin. Collectively, these findings indicate that trypsin is necessary for BCoV production in HRT-18G cells.

From these findings, 2.5 µg/mL trypsin was found to be the optimal concentration, so HRT-18G cells were infected with Rec-BCoV-Kakegawa-ZsGreen at a multiplicity of infection of 0.001 and cultured in the presence of 2.5 µg/mL trypsin. The ZsGreen signal was analyzed by microscopy. The number of ZsGreen positive cells peaked on day 4 and then spread (Fig. 2C and D).

We then analyzed the effect of 2.5 µg/mL trypsin on BCoV viral RNA levels and titers in culture supernatants. N RNA expression was predominantly higher in the presence of 2.5 µg/mL trypsin than in its absence (Fig. 2F). Similar to the increase in viral N RNA levels, the viral titer in the culture supernatant was predominantly higher in the presence of 2.5 µg/mL trypsin than in its absence (Fig. 2E). These results suggest that higher titers of BCoV can be recovered by adding the appropriate amount of trypsin (2.5 µg/mL) to BCoV cultures.

### Growth kinetics of recombinant BCoV-Kakegawa-ZsGreen virus

**Fig 3:**
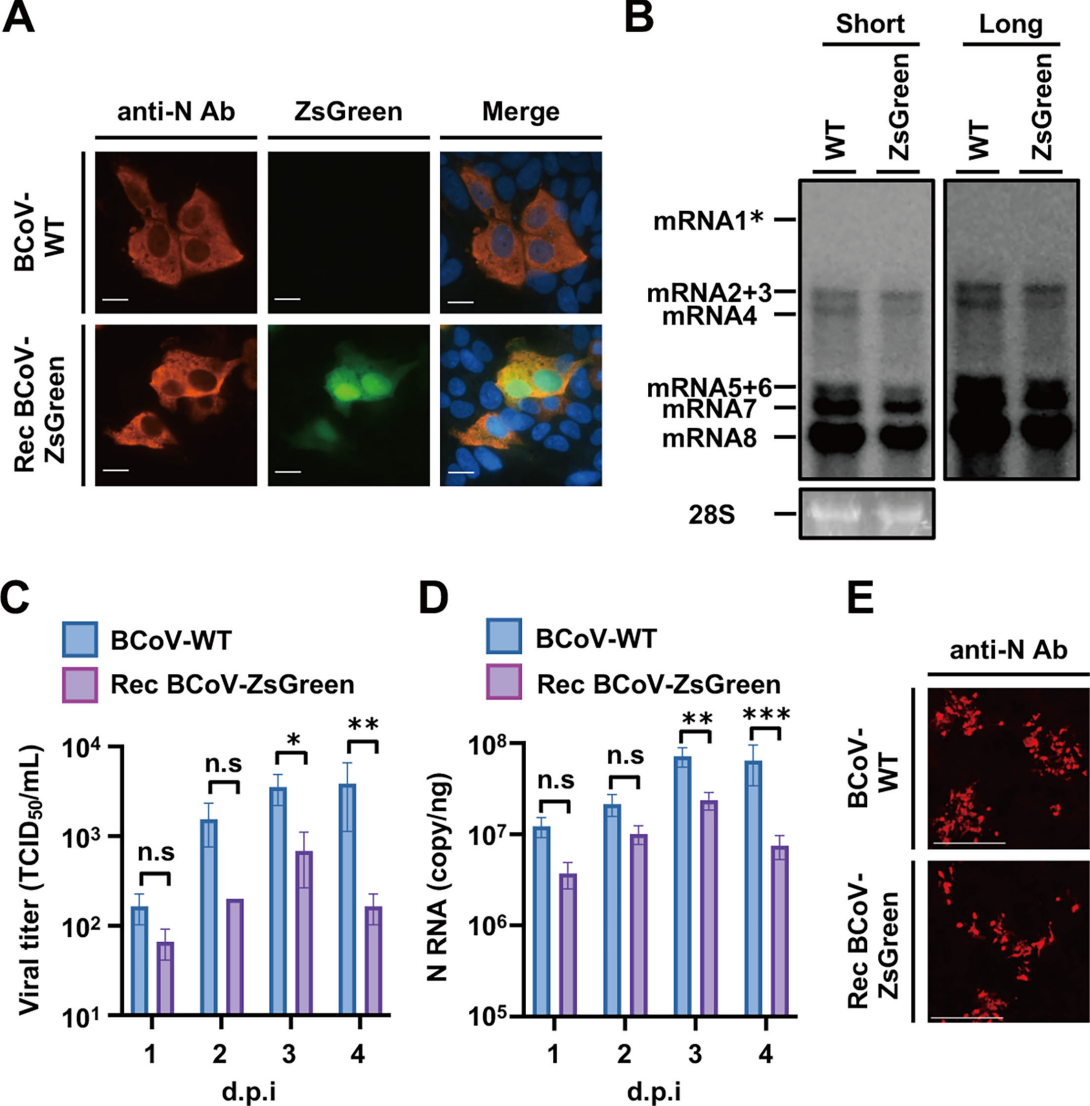
Virological properties of Rec-BCoV-Kakegawa-ZsGreen. (**A**) Subcellular localization of N and ZsGreen proteins in HRT-18G cells infected with Rec-BCoV-Kakegawa-WT. HRT-18G cells were infected with Rec-BCoV-Kakegawa-ZsGreen at an MOI of 0.001. At 3 d.p.i., the N protein was detected using an anti-OC43-N antibody and anti-rabbit IgG (H + L)-CF594 as primary and secondary antibodies, respectively. Scale bar = 10 µm. (**B**) Northern blot analysis detecting viral RNA in BCoV-Kakegawa-WT- or Rec-BCoV-Kakegawa-ZsGreen-infected HRT-18G cells. Total RNAs were extracted from HRT-18G cells infected with parental BCoV-Kakegawa-WT (WT) or Rec-BCoV-Kakegawa-ZsGreen (ZsGreen) and subjected to electrophoresis. The transferred viral RNAs were hybridized with DIG-labeled RNA targeting the 3′-UTR. * Expected migration of mRNA1. Short and Long represent the short and long exposures, respectively. (**C**) Comparison of infectious virus titers in the culture supernatants of Rec-BCoV-Kakegawa-ZsGreen and BCoV-Kakegawa-WT. HRT-18G cells were inoculated with bovine coronavirus at an M.O.I of 0.001 and incubated for 1–4 d.p.i. Viral titers in the culture supernatants were determined using IFA. ns, no statistically significant difference; d. p. i., days post-infection. The values represent the means ± SD from three independent experiments. *P < 0.05, **P < 0.01, and ***P < 0.001. (**D**) Comparison of viral N RNA accumulation in Rec-BCoV-Kakegawa-ZsGreen- and BCoV-Kakegawa-WT-infected HRT-18G cells. The expression of N RNA from bovine coronaviruses in HRT-18G cells was measured using a one-step real-time PCR. ns, no statistically significant difference; d.p.i., days post-infection. The values represent the means ± SD from three independent experiments. *P < 0.05, **P < 0.01. ***P < 0.001. (**E**) The BCoV-WT or Rec-BCoV-ZsGreen virus was inoculated into HRT-18G cells at an M.O.I of 0.0001. After incubating for 1 hour at 37°C, the cells were washed with PBS and overlaid with non-FBS DMEM containing 0.8% Seaplaque agarose. Three days post-infection, the N protein was detected using an anti-OC43-N antibody and an anti-rabbit IgG (H + L)-CF594 antibody as primary and secondary antibodies, respectively.

To analyze the virological characteristics of Rec-BCoV-Kakegawa-ZsGreen, a recombinant reporter virus, we demonstrated the localization of the N protein in HRT-18G cells infected with Rec-BCoV-Kakegawa-ZsGreen by IFA using the anti-OC43-N antibody of HCoV-OC43. This antibody recognizes the N protein. The N protein of BCoV-Kakegawa-WT was located in the cytoplasm of HRT-18G cells infected with BCoV-Kakegawa-WT (Fig. 3A). The N protein of Rec-BCoV-Kakegawa-ZsGreen was also found in the cytoplasm of HRT-18G cells infected with Rec-BCoV-Kakegawa-ZsGreen (Fig. 3A). The ZsGreen signal was located in both the nucleus and cytoplasm of HRT-18G cells infected with Rec-BCoV-Kakegawa-ZsGreen, and the cells positive for ZsGreen were consistent with the cells positive for the N protein (Fig. 3A). In the recombinant virus in which ORF2 protein was replaced by ZsGreen, the N protein was expressed in the same cytoplasm as in the parent strain.

Subsequently, the quantity of sub-genomic RNAs of the recombinant BCoV-Kakegawa-ZsGreen virus in infected HRT-18G cells was determined through Northern blotting utilizing a 3'UTR-specific ribonucleotide probe. Intracellular RNAs were extracted from infected HRT-18G cells and subsequently subjected to Northern blotting. This analysis revealed the presence of eight subgenomic RNAs in the samples infected with the Kakegawa strain, which is consistent with previous reports (27). In accordance with a previous study (27), the N subgenomic RNA was the most abundant among the eight subgenomic RNAs. Furthermore, the expression patterns of subgenomic RNA in HRT-18G cells infected with Rec-BCoV-Kakegawa-ZsGreen were analogous to those infected with BCoV-Kakegawa-WT (Fig. 3B). These observations indicate that the replacement of the ORF2 protein gene with the ZsGreen gene does not result in any alteration in the level of viral subgenomic RNA expressed.

As shown in Fig. 3B, subgenomic RNA expression was comparable between Rec-BCoV-Kakegawa-ZsGreen and the parental Kakegawa strain; therefore, we next analyzed N RNA expression in infected cells and the amount of infectious virus in the supernatant. On day 3, the amount of infectious virus in the supernatant of HRT-18G cells infected with the Kakegawa strain and that infected with the Rec-BCoV-Kakegawa-ZsGreen reached a value of 3.54 × 10^3^ TCID_50_/mL (Fig. 3C) and 6.86 × 10^2^ TCID_50_/mL (Fig. 3C), respectively. Furthermore, the amount of intracellular viral N RNA in HRT-18G cells infected with the Kakegawa strain reached a value of 7.18 × 10^7^ copies/ng on day 3 (Fig. 3D), while that in HRT-18G cells infected with Rec-BCoV-Kakegawa-ZsGreen was lower (Fig. 3D).

**Fig 4:**
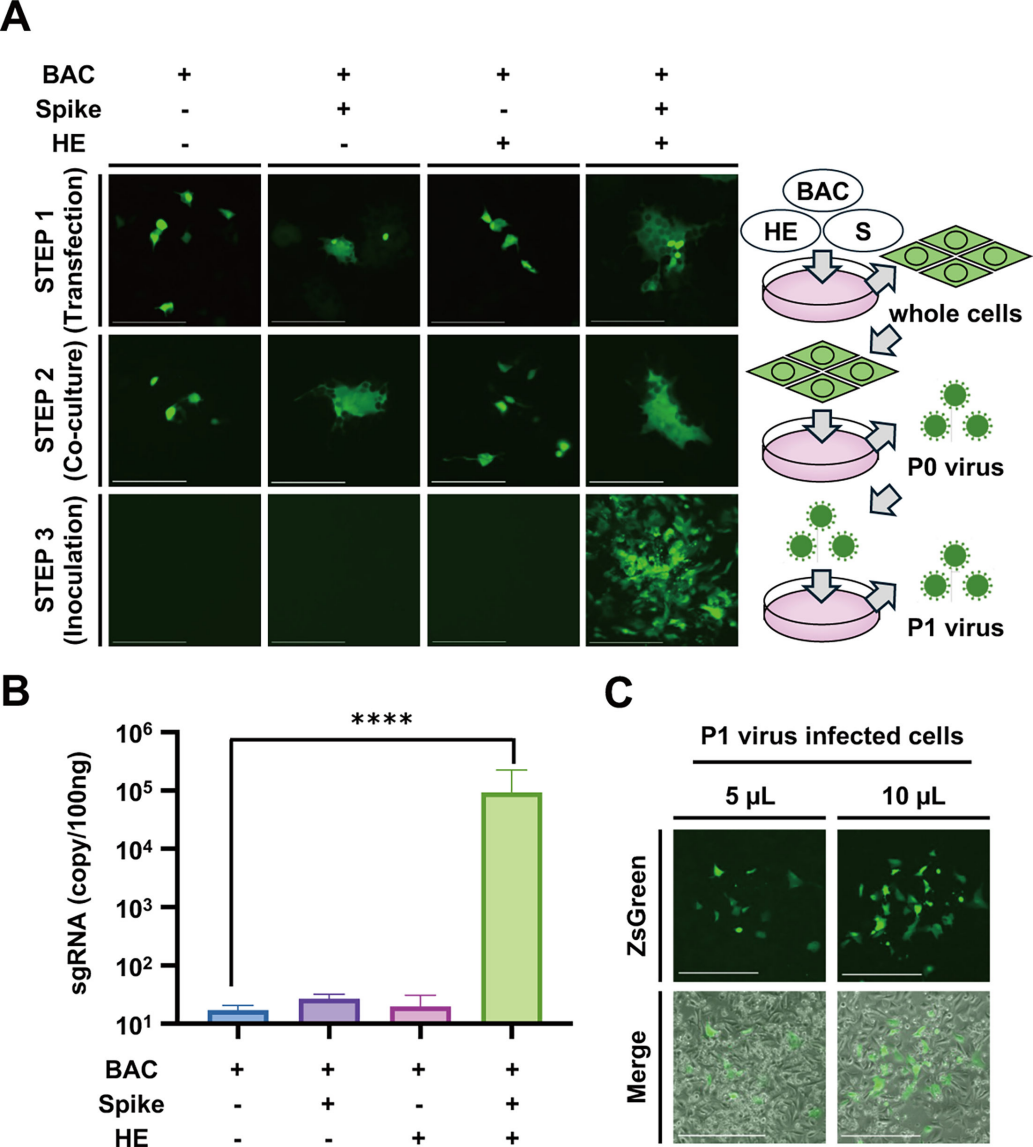
Necessity of co-expression of S and HE proteins in a reverse genetics system of BCoV. (**A**) Spread of ZsGreen fluorescence in indicated DNA-transfected 293T cells (top panels). Spread of ZsGreen fluorescence from indicated DNA-transfected 293T cells to co-cultured HRT-18G cells (middle panels). ZsGreen fluorescence in P0 virus-infected HRT-18G cells (bottom panels). ZsGreen fluorescence was detected using an M7000 microscope. Scale bar = 150 µm. (**B**) Expression of subgenomic N mRNA in P0 virus-infected HRT-18G cells. Intracellular RNAs extracted from P0-infected HRT-18G cells were subjected to real-time PCR using a specific set of primers and a probe to detect subgenomic N mRNA. The values represent the means ± SD from three independent experiments. (**C**) Naive HRT-18G cells were inoculated with 5 or 10 µL of P1 virus derived from co-cultured cells transfected with S and HE expression plasmids. Then, the ZsGreen signal was observed using an M7000 microscope.

We performed a focus-forming assay with an agar overlay to analyze Rec-BCoV-Kakegawa-ZsGreen viral replication. HRT-18G cells were infected with either Rec-BCoV-Kakegawa-ZsGreen or BCoV-Kakegawa-WT at an MOI of 0.0001. After 1 hour of incubation, the medium was removed, and the cells were washed with PBS. Then, non-FBS DMEM containing 0.8% Seaplaque agar was added. The foci were visualized using anti-OC43 N and anti-rabbit IgG CF594 antibodies under an M7000 imaging system. Fig. 4E shows that the foci size in HRT-18G cells infected with Rec-BCoV-Kakegawa-ZsGreen was smaller than that in cells infected with BCoV-Kakegawa-WT. These results indicated that Rec-BCoV-Kakegawa-ZsGreen exhibited a slightly diminished viral growth compared to the parental strain. However, the recombinant virus retained properties of its parental strain.

### Demand for S and HE proteins in the production of recombinant BCoV

To analyze the requirements for S and HE proteins during recombinant BCoV production, a combination experiment was performed, as shown in Fig. 4A. 293T cells were transfected with the indicated DNA and treated with trypsin (Fig. 4A, step1) and then co-cultured with HRT-18G cells in the presence of trypsin (Fig. 4A, step2). The virus was harvested from co-cultured cells by freeze-thawing and used to infect HRT-18G cells (Fig. 4A, step 3). In step 3, ZsGreen-positive cells were observed only when S and HE protein expression plasmids were supplied (Fig. 4A).

Subgenomic N mRNA levels were measured by qPCR using step 3 samples, and 9.22 × 10^4^ copies/100 ng values were observed only in samples delivered with two plasmids: the S and HE protein expression plasmids (Fig. 4B).

Although the infected cells were washed three times with PBS after incubation with the supernatant from the co-cultured sample, the S and HE proteins may have been contaminated in step 3. To eliminate the possibility of carryover of the S and HE proteins, the P1 virus obtained was inoculated into naïve HRT-18G, and the ZsGreen signal was examined under an M7000 microscope imaging system. As shown in Fig. 4C, a ZsGreen signal was clearly observed in samples infected with 5 or 10 µL P1 virus. These results suggest that expression of S and HE proteins may be necessary for the co-culture step in the generation of recombinant viruses.

These results indicate that the co-expression of S and HE proteins during BCoV recombinant virus generation is important for efficient recombinant virus production.

### Authentic recombinant BCoV recovery

**Fig 5:**
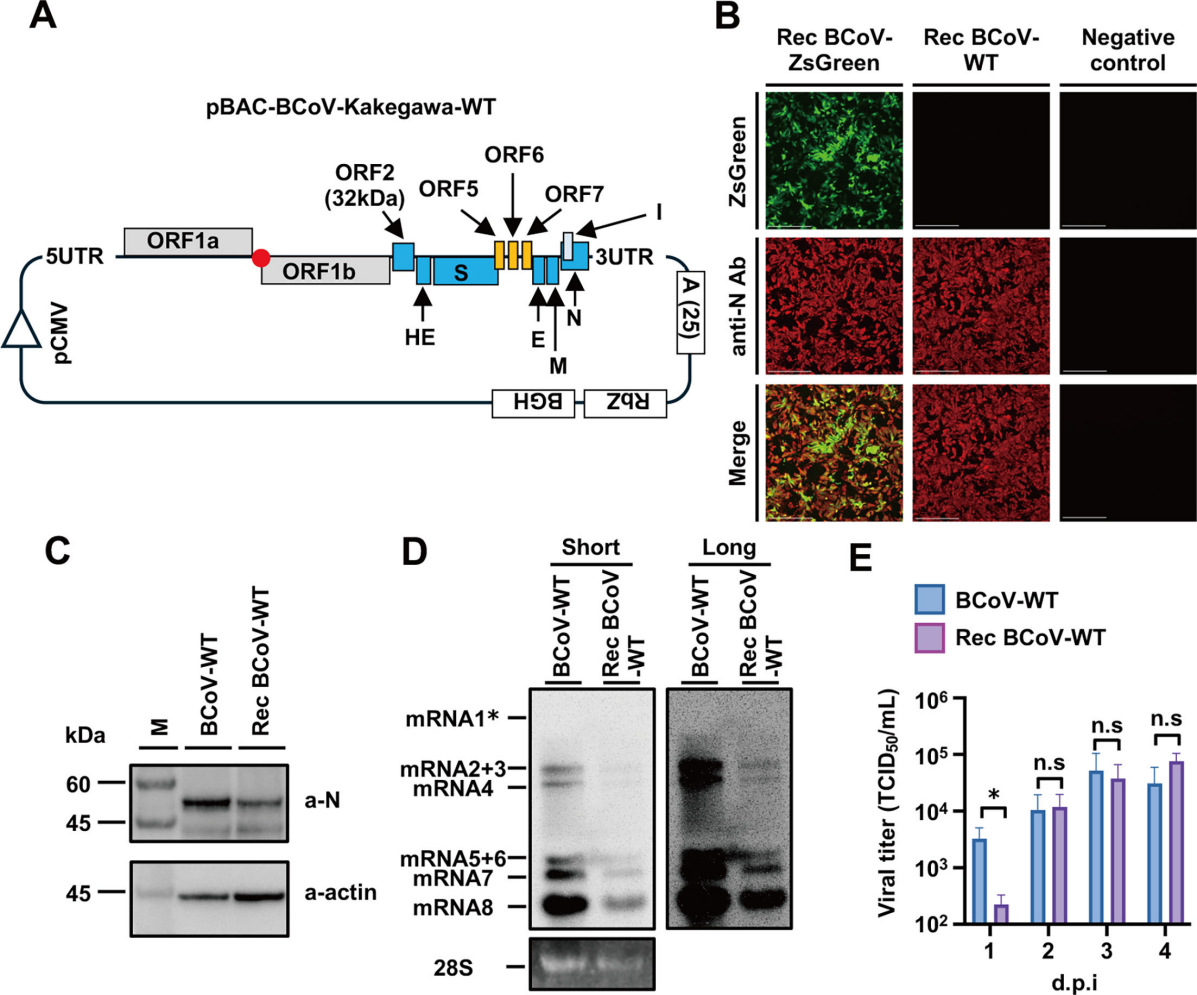
Recombinant BCoV recovery. (**A**) Schematic representation of the construction of infectious cDNA clones of the authentic Kakegawa strain of bovine coronavirus. pCMV: cytomegalovirus promoter, 5UTR: 5' untranslated region, ORF1a, ORF1b, ORF2, ORF5, ORF6, and ORF7: open reading frame 1a, 1b, 2, 5, 6, and 7, HE: hemagglutinin esterase, S: spike, M: matrix, E: envelope, N: nucleoprotein, I: internal protein, 3UTR: 3' untranslated region, A(25): synthetic 25 nt polyA, RbZ: ribozyme sequence of HDV, BGH: bovine growth factor polyadenylation signal. (**B**) N-protein expression in HRT-18G cells infected with recombinant authentic BCoV. HRT-18G cells were infected with BCoV at an M.O.I of 0.001, permeabilized with 1% Triton-X in PBS. After 72 hours, the infected cells were fixed with 4% paraformaldehyde and permeabilized with 1% Triton-X in PBS. N protein was detected using anti-OC43-N antibody and anti-rabbit IgG (H + L)-CF594 antibody as primary and secondary antibodies, respectively. N protein-positive cells were observed using an M7000 microscope. (**C**) Detection of N protein in HRT-18G cells infected with BCoV-WT or Rec-BCoV-WT. Lysates of infected HRT-18G cells were prepared 48 hours post-infection and subjected to Western blotting using an anti-OC43-N polyclonal antibody ((a-N) or anti-actin antibody (a-actin) as the primary antibody. M, molecular weight marker. (**D**) Northern blot analysis detecting viral RNA in BCoV-Kakegawa-WT- or Rec-BCoV-Kakegawa-WT-infected HRT-18G cells. Total RNAs were extracted from HRT-18G cells infected with parental BCoV-Kakegawa-WT or Rec-BCoV-Kakegawa-WT and subjected to electrophoresis. The transferred viral RNAs were hybridized with DIG-labeled RNA targeting the 3′-UTR. * Expected migration of mRNA1. Short and Long represent the short and long exposures, respectively. (**E**) Comparison of infectious virus titers in the culture supernatants of Rec-BCoV-Kakegawa-WT and BCoV-Kakegawa-WT. HRT-18G cells were inoculated with bovine coronavirus at an M.O.I of 0.001 and incubated for 1–4 d.p.i. Viral titers in the culture supernatants were determined using IFA. ns, no statistically significant difference; d. p. i., days post-infection. The values represent the means ± SD from three independent experiments. *P < 0.05.

Next, we attempted to rescue Rec-BCoV-Kakegawa-WT, which has an ORF2 gene instead of the ZsGreen gene, because we optimized the protocol using Rec-BCoV-Kakegawa-ZsGreen. First, we constructed pBAC-BCoV-Kakegawa-WT using pBAC-BCoV-Kakegawa-ZsGreen as the template (Fig. 5A). Then, 8 µg of pBAC-BCoV-Kakekgawa-WT was transfected into 293T cells with pCAG-BCoV-S-Δ17AA and pCAG-Igk leader-BCoV-HE-FLAG plasmids. After 2 days of incubation, the medium of transfected cells was changed to FBS-free DMEM with 5 µg/mL of trypsin. After 8 h of trypsin treatment, the cells were overlaid with HRT-18G cells. After 4 days of co-culture, the P0 virus was prepared from the co-cultured cells by freezing and thawing.

To confirm whether Rec-BCoV-Kakegawa-WT was recovered from cultured cells, the N protein was detected in P0 virus-infected cells using IFA, as shown in Fig. 5B. The number of N protein-positive cells infected with Rec-BCoV-Kakegawa-WT was similar to that of cells infected with Rec-BCoV-Kakegawa-ZsGreen, as shown in Fig. 5B. These results indicated the successful generation of a recombinant virus with a full-length BCoV genome.

To analyze the virological future of Rec-BCoV-Kakegawa-WT, the expression level of the N protein in infected cells was detected. As shown in Fig. 5C, the expression level of the N protein in HRT-18G cells infected with Rec-BCoV-Kakegawa-WT was slightly lower than that in cells infected with BCoV-Kakegawa-WT. Northern blotting revealed that the expression of subgenomic RNA in HRT-18G cells infected with Rec-BCoV-Kakegawa-WT was lower than that in cells infected with BCoV-Kakegawa-WT, as shown in Fig. 5D. Although the expression levels of the N protein and subgenomic RNAs were slightly lower in Rec-BCoV-Kakegawa-WT-infected HRT-18G cells (Fig. 5C and D), the virus titer in the culture supernatant of Rec-BCoV-Kakegawa-WT-infected cells was comparable to that of BCoV-Kakegawa-WT-infected cells, as shown in Fig. 5E.

As shown in Table S2, the Rec-BCoV-Kakegawa-WT viral genome sequence was not identical to the reference sequence. This difference may affect the expression of N and subgenomic RNAs of Rec-BCoV-Kakegawa-WT.

The results suggest that the Rec-BCoV-Kakegawa-WT maintains the viral characteristics of the parental virus.

## DISCUSSION

In this study, we successfully generated a recombinant BCoV by utilizing an infectious BAC DNA clone from infected cells that expressed the BCoV S and HE proteins. Generally, the co-expression of viral proteins, with the exception of the N protein, does not require reverse genetics in coronaviruses. The *in vitro*-transcribed RNA of an infectious coronavirus RNA is co-transfected with N-expressing RNA into BHK cells or suitable cell lines (8, 9). In the case of HCoV-OC43, which is genetically close to BCoV, it was reported that the infectious virus was successfully recovered by simply introducing BAC-based infectious DNA into cultured cells (16), while in the case of yeast-based infectious DNA, the infectious virus was successfully recovered by co-expression of the infectious DNA and N protein (24).

In our BAC-based reverse genetics system for BCoV, two viral proteins, S and HE, are required to rescue both Rec-BCoV-Kakegawa-ZsGreen and Rec-BCoV-Kakegawa-WT. The HE protein of BCoV is necessary to destroy endogenous viral receptors, thereby enhancing the efficiency of viral budding or assembly (26, 28). The HE protein of coronaviruses is a unique structural protein, and only a few coronaviruses, including BCoV and human coronavirus OC43, possess this characteristic (4, 29). It has been reported that expression of the BCoV HE protein with an additional secretory signal is necessary for the generation of VSV-based pseudotyped viruses that express the BCoV S protein (26). Expression of the HE protein alone was insufficient to recover the Rec-BCoV-Kakegawa-ZsGreen virus, but when the HE protein was expressed together with the S protein, the recombinant virus was successfully recovered from cultured cells (Fig. 4).

The BCoV utilizes glycan-based receptors carrying 9-O-acetylated sialic acid (9-O-Ac-Sia) (28, 30, 31). Although N protein expression is important for the generation of recombinant HCoV-OC43 virus (24), it can still be recovered without N protein expression (16). Therefore, S protein co-expression was used to generate recombinant BCoV. As shown in Fig. 4, the ZsGreen-positive region in the transfected 293T cells expanded upon transfection with the S protein expression plasmid. The S protein expressed in the transfected cells may have bound to 9-O-Ac-Sia on the cell membrane surface of the 293T cells, causing cell fusion. This cell fusion could potentially provide more host cell-derived resources for viral replication. This could be a new method for generating recombinant coronaviruses that replicate poorly in cultured cells, such as field isolates, although there are no reports on the need to supply both S and HE proteins in the generation of recombinant coronaviruses.

 In cultured cells, some coronaviruses, including porcine epidemic diarrhea virus, require a suitable concentration of trypsin to enhance viral entry (32–40). However, for BCoV, the trypsin requirement for cultured cells is unknown. Several studies have reported the addition of trypsin to cells cultured with BCoV (41–43). We used Rec-BCoV-Kakegawa-ZsGreen to clarify the role of trypsin in the BCoV culture. Despite significant trypsin-induced toxicity in HRT-18G cells, the virus titer in the supernatant of infected HRT-18G cells was increased in a time-dependent manner for up to 3 days post infection in the presence of 2.5 µg/mL of trypsin (Fig. 3). In general, when field isolates of bovine coronavirus are isolated and cultured, the viral titer is expected to be low. Therefore, the key to isolating and culturing field isolates of bovine coronavirus is to recover the virus with as high a titer as possible. Therefore, we believe that our optimization of trypsin concentration during bovine coronavirus culture will help dramatically accelerate the virological analysis of bovine coronaviruses.

In this study, infectious BAC DNA was constructed by replacing the ORF2 gene region with the ZsGreen gene within the BCoV genome. A region homologous to the ORF2 region of BCoV is present in the genome of HCoV-OC43 as the ns2 gene. The ns2 protein of HCoV-OC43 is known to localize to the cytoplasm (44) and to repress the RNaseL pathway via the 2',5'-phosphodiesterase activity (45). The function of the ORF2 protein remains to be elucidated; however, it has been postulated that it is not essential for viral replication. The growth of Rec-BCoV-Kakegawa-ZsGreen, in which the ORF2 gene was substituted with the ZsGreen gene, in cultured cells did not differ significantly from that of the parental strain, BCoV-Kakegawa-WT, for up to 2 days after infection (Fig. 3C and D). However, the peak titers were significantly lower than those of BCoV-Kakegawa-WT. This observation indicates that the ORF2 viral protein, although dispensable for viral replication, potentially exerts a direct or indirect influence on viral replication in the subsequent stages of the replication cycle. The ZsGreen-positive cells observed in the Rec-BCoV-Kakegawa-ZsGreen-infected cells in this study exhibited concurrence with N protein-positive cells. Conversely, ZsGreen-negative cells were identified as N protein-positive cells using IFA. It is plausible that these N protein-positive and ZsGreen-negative cells do not express sufficient levels of ZsGreen protein during the initial stages of infection. Furthermore, the fluorescence intensity of N protein expression in these cells was weaker than that in the other N protein-positive cells. Collectively, these observations suggest the potential absence of the ZsGreen gene from the viral gene of the recombinant virus. In the future, it will be necessary to create recombinant viruses that stably express ZsGreen.

The Kakegawa strain of BCoV used to generate the recombinant BCoV in this study is a laboratory strain. Interestingly, a field isolate with a four amino acid insertion or mutation in the HE protein has been previously reported (46, 47). There are also reports suggesting that the loss of esterase activity in the HE protein of HCoV-OC43 is necessary for human adaptation (48). In the future, the established BCoV genetic manipulation system will be an important tool for analyzing the function of the BCoV HE protein and may further elucidate the mechanism of host jumping to humans by the HE protein.

The objective of this study was to create recombinant viruses with reporter genes, such as ZsGreen, owing to the limitations of CPE in cultured cells during bovine coronavirus infection. The presence of the CPE is crucial for the success of viral recovery during the creation of a recombinant virus. However, in cases where viruses exhibit weak CPE in cultured cells, it is challenging to ascertain the presence or absence of an infectious virus at the time of virus generation based solely on CPE observations. We engineered recombinant viruses by utilizing infectious BAC DNA, in which the viral gene segment was substituted with an ORF2 gene. Finally, we succeeded in recovering Rec-BCoV-Kakegawa-WT, which contained the entire gene. We used pBAC-BCoV-Kakegawa-ZsGreen as a tracer to rescue Rec-BCoV-Kakegawa-WT from the infectious DNA. As Rec-BCoV-Kakegawa-WT did not induce a strong CPE, it was useful to use Rec-BCoV-Kakegawa-ZsGreen in the same way when making BCoV.

In conclusion, we successfully obtained recombinant BCoV Kakegawa from infectious BAC DNA using co-expression of both S and HE proteins.

## MATERIALS AND METHODS

### Cells and viruses

293T (human embryonic kidney) and HRT-18G (human rectal tumor cells) were maintained in DMEM (Nacalai Tesque, Kyoto, Japan) supplemented with 10% FBS (Biosera, Cholet, France). The BCoV Kakegawa strain was propagated in HRT-18G cells, and the titer was measured in HRT-18G cells at TCID50.

### Plasmid DNA and BAC DNA construction

To generate the BCoV S protein expressing plasmid with a 17AA deletion in the C-terminus, the expressing plasmid (pCAG-BCoV-S-Δ17AA) was cloned into the pCAGGS-multiple cloning site (MCS) vector. To produce the BCoV HE protein, a plasmid with an Igk leader and FLAG tag sequence was constructed and cloned into pCAGGS-MCS according to a previous report (26).

To generate infectious DNA of BCoV (Kakegawa strain) using BAC, the RNA was extracted from HRT-18G cells infected with BCoV, and then the cDNA of BCoV was synthesized using Superscript IV (Thermo Fisher Scientific, Waltham, MA, USA) with a random primer from the extracted RNAs. The full-length sequence of BCoV was amplified using the prepared cDNA as a template, GXL polymerase (Takara, Shiga, Japan), and primers overlapping 40 bases, resulting in a fragment of the full-length sequence of BCoV approximately every 5,000 base pairs. The backbone BAC DNA was amplified using GXL polymerase (Takara) and overlapping primers of 40 bp containing the cytomegalovirus promoter, 25 nt polyA sequence, and HDV ribozyme.

PCR fragments were amplified using the primers shown in Table S1 with 40-base overlaps at both ends of each fragment, using Takara GXL polymerase. Subsequently, the target-length bands were extracted from the gel using a FastGene Gel/PCR Extraction Kit (Nippon Genetics, Tokyo, Japan). DNA assembly was then performed using a Gibson Assembly Kit (Biosearch Technologies, Hoddesdon, United Kingdom). Following the manufacturer’s instructions, the DNA was adjusted to 0.009 pmol, mixed to 5 µL, and then 5 µL of reaction solution A was added. A 3′ end chew-back reaction was performed at 37°C for 5 minutes, followed by an inactivation reaction at 75°C for 20 minutes and an annealing reaction at 60°C for 30 minutes. Then, 10 µL of solution B was added, followed by a 15 minute repair reaction at 45°C. The reaction mixture was then precipitated with ethanol and dissolved in 10 µL of DDW.

Twenty microliters of *E. coli* was added to 10 µL DNA, and then transformation was performed at 1,400 V using an Eppendorf Eporator. After a 1 hour recovery period, the colonies were inoculated onto LB plates containing chloramphenicol and incubated at 37°C until colonies appeared. All colonies containing the fragments were screened using direct PCR. The complete sequence of the extracted DNA was confirmed by Sanger sequencing (Eurofins Scientific, Tokyo, Japan), and the infectious BAC DNA was designated as pBAC-BCoV-Kakegawa-WT.

### Red/ET recombination

To create the reporter virus, the ORF2 gene sequence of BCoV was replaced with a ZsGreen sequence using a Red/ET recombination system counter-selection BAC modification kit (Gene Bridges, Heidelberg, Germany).

First, the selection marker (rpsL-neo-DNA), comprising the kanamycin resistance (neomycin) and streptomycin susceptibility (rpsL) genes, was amplified using PCR and the GXL enzyme. The viral genome region encoding the ORF2 protein was used as a template. PCR primers (5ʹ-AAGAAGTTTTTGTTGGTGACAGTATGGTTAATGTAATCTAAACTTTAAGAGGCCTGGTGATGATGGCGGGATCG-3ʹ and 5ʹ-CTAAATTTTGACAAGACTTACGGAAATAAGAACTTTTCCTGTAGACATGTTCAGAAGAACTCGTCAAGAAGGCG-3ʹ) containing a 50-base homologous region necessary for homologous recombination were then employed. The pRedET plasmid, necessary for homologous recombination, was then transformed into *E. coli*. The amplified PCR fragment was then introduced into the *E. coli* using an Eppendorf Eporator at 2,000 V. Following the instructions in the attached documents, positive clones were screened using kanamycin and streptomycin.

A second homologous recombination was then performed to replace the selection marker region with the ZsGreen gene. ZsGreen DNA was first amplified using PCR with GXL enzyme and PCR primers (5ʹ-AAGAAGTTTTTGTTGGTGACAGTATGGTTAATGTAATCTAAACTTTAAGAATGGCCCAGTCCAAGCACGGCCTGA-3ʹ and 5ʹ-CTAAATTTTGACAAGACTTACGGAAATAAGAACTTTTCCTGTAGACATGTTCAGGGCAAGGCGGAGCCGGAGGCG-3ʹ) containing the necessary 50-base homologous region for homologous recombination. The amplified DNA was then introduced into the positive *E. coli* clones, which were identified by screening, using an Eppendorf Eporator at 2000 V. Positive clones were identified by direct PCR, and the target DNA was purified. This construct was designated as pBAC-BCoV-Kakegawa-ZsGreen. Sequence analysis was performed by Eurofins Scientific (Tokyo, Japan) to confirm substitutions.

### Recovering recombinant virus

The recovery of recombinant BCoV was achieved through the seeding of 293T cells at a density of 4 × 10⁵ cells/mL in a 6-well plate (Grainer Bio-One, Frickenhausen, Germany). Following a 24 hour incubation period, 8 µg of pBAC-BCoV-Kakegawa-ZsGreen, in conjunction with 1 µg of pCAG-BCoV-S-Δ17AA plasmid, and 1 µg of pCAG-Igk leader-BCoV-HE-FLAG plasmid, were introduced into the cells. 293T cells were transfected with XtreamGene9 (Roche, Basel, Switzerland), followed by culturing the transfected 293T cells until ZsGreen signals were observed under a microscope. Following confirmation of the ZsGreen-positive cells, treatment with 5 µg/mL of trypsin (T4799-5G, Sigma-Aldrich) was initiated for a duration of 8 h. Subsequently, all co-cultured cells were overlaid onto ZsGreen-positive 293T cells. The ZsGreen-positive cells then expanded, and the co-cultured cells were collected and subjected to freeze-thaw cycles to obtain the recombinant ZsGreen virus as a P1 virus.

### Titration

The 50% tissue culture infectious dose (TCID50) method was used to determine the infectious titer for each virus. Briefly, HRT-18G cells were seeded in 96-well plates (Greiner) and cultured overnight at 37°C. Viruses were serially diluted 10-fold using DMEM without FBS. The diluted viruses were inoculated into HRT-18G cells and incubated at 37°C for 3 days. After incubation, the infected cells were fixed with 4% paraformaldehyde (Nacalai Tesque) and immunostained using an anti-OC43 N peptide antibody (homemade) and anti-rabbit IgG (H + L)-CF594 antibody (Sigma, St. Louis, MO, USA) as secondary antibodies. Viral foci were observed using an M7000 imaging system microscope (Thermo Fisher Scientific), and TCID50 was calculated using the Reed-Muench algorithm.

### Quantitative RT-PCR

Total RNA was extracted from the infected cells using a PureLink RNA Mini kit (Thermo Fisher Scientific) according to the manufacturer’s instructions. To quantify the viral RNAs, a one-step probe qPCR method was performed using the THUNDERBIRD Probe One-step qRT-PCR Kit (TOYOBO, Osaka, Japan). Two PCR primers targeting the BCoV N gene (5′- CGATGAGGCTATTCCGACTAGGT-3′; 5′- CCTTCCTGAGCCTTCAATATAGTAACC-3′) and one probe (5′-[FAM] TCCGCCTGGCACGGTACTCCCT [TAM]-3′) were applied (49, 50). To detect sub-genomic N mRNA, two PCR primers targeting the BCoV sub-genomic N gene (5′- TCCCGCTTCACTGATCTCTT-3′; 5′- AGGATGCCATTACCAGAACG-3′) and one probe (5′-[FAM] TCCAGTAGTAGAGCGTCCTCTGGA [TAM]-3′) were applied. A StepOnePlus Real-Time PCR system (Thermo Fisher Scientific) was used for all the RT-qPCR experiments. To measure the number of copies, *in vitro* transcribed RNA was used as the standard.

### Immunoblotting

The transfected 293T cells were lysed in RIPA buffer (Nacalai Tesque, Kyoto, Japan). After centrifugation at 10,000 × *g*, supernatants were collected and mixed with 2 × sample buffer (0.1 M Tris-HCl pH 6.8, 4% sodium dodecyl sulfate [SDS], 20% glycerol, 0.004% bromophenol blue, and 10% 2-mercaptethanol). The samples were boiled. Proteins were separated by 5%–20% SDS-polyacrylamide gel electrophoresis (SDS-PAGE) (e-Pagel, ATTO, Tokyo, Japan). The electrophoresed proteins were transferred onto polyvinylidene difluoride membranes using Q Blot Kit M (ATTO). Anti-OC43-S1 polyclonal (BS80272; Bioworld Technology) and anti-DYKDDDK monoclonal (M185-3L; MBL) antibodies were used as primary antibodies. Anti-mouse IgG(H + L)-HRP (SAB3701066; Sigma-Aldrich) and anti-rabbit IgG(H + L)-HRP (SAB3700878; Sigma-Aldrich) were used as secondary antibodies. ChemiLumi One Super (Nacalai Tesque) and LuminoGraph I (ATTO) were used for visualization. The antibody against S used in this study is a peptide antibody against the S of HCoV-OC43. The amino acids recognized by this peptide antibody also perfectly match those of the S of BCoV.

### Northern blotting

The intracellular RNA of infected HRT-18G cells was extracted using a PureLink RNA Mini Kit (Thermo Fisher Scientific) according to the manufacturer’s instructions and stored at −80°C until use. The RNA samples were mixed with 2 × loading dye (New England Biolabs, Ipswich, MA, USA). After heating at 65°C for 5 min, the RNA samples were electrophoresed on a 1.2% denaturing agarose gel and transferred onto a positively charged nylon membrane (Roche, Basel, Switzerland). Northern blot analysis was performed using a digoxigenin (DIG) wash and block buffer set and a DIG luminescence detection kit (Roche). The DIG-labeled riboprobe to detect 3' UTR of BCoV was generated using a DIG RNA labeling kit (SP6/T7) (Roche), as described previously (25, 27, 51).

### Immunofluorescence assay

The HRT-18G cells were infected with BCoV. Following incubation at 37°C, infected cells were fixed with 4% paraformaldehyde for 10 min and permeabilized with phosphate-buffered saline containing 1% Triton-X for 10 min. Anti-OC43-N peptide antibody (Scrum, Tokyo, Japan) was used as the primary antibody, and anti-rabbit IgG(H + L)-CF594 (Sigma, St. Louis, MO) was used as the secondary antibody. The cells were examined using an EVOS M7000 imaging system (Thermo Fisher Scientific), as described previously (52).

### Focus forming assay

The BCoV-WT or Rec-BCoV-ZsGreen virus was inoculated into HRT-18G cells at an MOI of 0.0001. After incubating for 1 hour at 37°C, the cells were washed with PBS and overlaid with non-FBS DMEM containing 0.8% Seaplaque agarose. Three days post-infection, the N protein was detected using an anti-OC43-N peptide antibody and an anti-rabbit IgG (H + L)-CF594 antibody as primary and secondary antibodies, respectively. The N-positive foci were observed using an EVOS M7000 imaging system (Thermo Fisher Scientific).

### Statistical analysis

Student’s *t*-test and one- and two-way ANOVA were conducted to determine the statistical significance using GraphPad Prism version 10 (GraphPad Software, San Diego, CA, USA). Statistical significance was set at *P* < 0.05.

## Data Availability

The data that support the findings of this study are available from the corresponding author, W.K., upon reasonable request.

## SUPPLEMENTAL MATERIAL

Supplemental tables jvi.01027-25-s0001.pdf Tables S1 to S3.

ASM does not own the copyrights to Supplemental Material that may be linked to, or accessed through, an article. The authors have granted ASM a non-exclusive, world-wide license to publish the Supplemental Material files. Please contact the corresponding author directly for reuse.
