# Supplementary material for: Expression of spike and hemagglutinin-esterase proteins is necessary to recover infectious recombinant bovine coronavirus
Source: J Virol. 2025 Aug 11;99(9):e01027-25. doi: 10.1128/jvi.01027-25 (PMC12456148; doi:10.1128/jvi.01027-25)
Supplement: Supplemental tables — Tables S1 to S3. [file jvi.01027-25-s0001.pdf]

Supplementary Information

Table S1. Primer sets for generation of pBAC-BCoV-Kakegawa-WT

Fragment	Direction	Sequence (5' to 3')
F1	Forword	GATTGTGAGCGATTTGCGTGCGTGCATCCCGCTTCACTGATCTCTTGT
F1	Reverse	GTTTCGTGACATTAACTCCATCACAA
F2	Forword	CTAGGAAATGTGTTTTGTGATGGAGTTA
F2	Reverse	ATTACCATAGGTAACACTGACAATACATGG
F3	Forword	TAAGGTAGAACCATGTATTGTCAGTGT
F3	Reverse	ATGAAACATTCTGCCAGTCATAGTACTA
F4	Forword	TGTTTCCATACTTAGTACTATGACTGGC
F4	Reverse	ATTAACATTAACAACCTTACTCACACAGT
F5	Forword	CTTAATCTTAACTGTGTGAGTAAGGTTGTT
F5	Reverse	AAAAACAGATTGTTCTGTAAAACCAAATCTC
F6	Forword	TTGGAATAGGAGATTTGGTTTTACAGAAC
F6	Reverse	TTATATTTCTGAGGTGTCTTCAGTATAGGG
F7	Forword	GATGGATGAGCCCTATACTGAAGACACCTCAGAAATATAAGAGAATGAACCTTATG TCGGCACCT
F7	Reverse	TCCCTTAGCCATCCGAGTG
pCMV+pA+ Rz+BGH	Forword	CATCCGAAGGAGGACGTCG
pCMV+pA+ Rz+BGH	Reverse	TCAGTGAAGCGGGATGCACGCACGCAAATCGCTCACAATCACGGTTCACTAAACG AGCTCTGCTT

Supplementary Information

Table S2. Analysis of mutations in recombinant BCoV-WT viral genome

Mutation no.	Nucleotide Position	Gene	Nucleotide mutation	Amino acid mutation
1	408	1a (nsp1)	T → C	synonymous
2	1031	1a (nsp1)	G → A	R → K
3	1392	1a (nsp1)	T → A	H → Q
4	2424	1a (nsp2)	T → C	synonymous
5	2700	1a (nsp2)	T → C	synonymous
6	3231	1a (nsp3)	C → T	synonymous
7	3248	1a (nsp3)	C → A	A → D
8	3752	1a (nsp3)	G → A	G → E
9	7746	1a (nsp3)	T → C	synonymous
10	9160	1a (nsp4)	C → T	L → F
11	12887	1a (nsp9)	C → T	S → F
12	14186	1b (nsp12)	C → T	synonymous
13	15756	1b (nsp12)	A → G	M → V
14	16207	1b (nsp13)	C → T	A → V
15	18924	1b (nsp14)	T → C	C → R

16	21486	1b (nsp16)	G → A	V → I
17	23759	S	C → T	T → I
18	24398	S	A → C	Y → S
19	25231	S	G → A	D → N
20	26593	S	T → A	S → T
21	28522	E	C → G	L → V
22	28641	E	C → T	synonymous
23	28729	M	C → G	T → S
24	28806	M	A → G	I → V
25	28820	M	T → C	synonymous
26	28898	M	C → T	synonymous
27	28961	M	C → T	synonymous
28	29231	M	C → T	synonymous
29	29241	M	T → C	F → L
30	29279	N	T → C	synonymous
31	29380	N	T → A	I → N
32	29381	N	A → T	I → N
33	29542	N	C → T	A → V
34	29618	N	A → G	synonymous
35	29624	N	G → A	synonymous
36	29627	N	T → G	D → E
37	29675	N	G → A	synonymous
38	29720	N	T → C	synonymous
39	29751	N	C → T	synonymous
40	29819	N	T → C	synonymous
41	29882	N	C → T	synonymous
42	30558	N	A → G	N → D

Table S2. Analysis of mutations in the recombinant BCoV-WT viral genome.

The cDNA was synthesized using the SuperScript IV enzyme from the extracted RNA of the P1 sample of the Rec-BCoV-WT virus. To synthesize the PCR fragments of the Rec-BCoV-WT viral

cDNA, the primer sets shown in Table S3 were used. Each PCR fragment was purified and subjected to Sanger sequencing (Eurofins Scientific). GenBank database number AB354579.1 was used as a reference sequence.

Supplementary Information

Table S3. Primer sets for Sanger sequencing

Primer name	Sequence
BCoV TCG25 Left1	CCCGCTTCACTGATCTCTTGT
BCoV TCG25 Right6	AGCAAGAACTTCCTCTCCAGC
BCoV TCG25 Left7	GCTGTGGTGGTTGATGCCAT
BCoV TCG25 Right12	CTGTAGCTGTTGGCCACTCA
BCoV TCG25 Left13	ACTCAGCGTATTATTAAAGCCCA
BCoV TCG25 Right18	GCACAAGTCCTTCTCGCAAC
BCoV TCG25 Left19	TTGCAATGGCCGATGGTAGT
BCoV TCG25 Right24	AGAACGCGCCTCATCAAGAT
BCoV TCG25 Left25	GCTGTGGATAGCAAGTGCCT
BCoV TCG25 Right30	CAGGAACACCACGTGTAGCT
BCoV TCG25 Left31	AGTGCTAAGAATAGAGCCCGC
BCoV TCG25 Right36	ACAGCAGAATCTCCAATACCAAG
BCoV TCG25 Left37	TGGTTACCACCCAGCTCAC
BCoV TCG25 Right42	ACCCAGAGCTAACTTGTCGC
BCoV TCG25 Left43	AGTGTCGCTACATATTTTGGGGA
BCoV TCG25 Right48	ACCCAAAATGGTAGTATGTGGT
BCoV TCG25 Left49	ACCACCTTTTCTTTCTGATTTTACT
BCoV TCG25 Right54	ACCATTACCACAAAAATTTATCCTAGA
BCoV TCG25 Left55	CGTCTTACCGCTCTTAATGCT
BCoV TCG25 Right60	ACACCAGAGGTAGGGGTTCT
